# Quantification of time delay between screening and subsequent initiation of contact isolation for carriers of extended-spectrum beta-lactamase (ESBL)–producing Enterobacterales: A post hoc subgroup analysis of the R-GNOSIS WP5 Trial

**DOI:** 10.1017/ice.2022.285

**Published:** 2023-09

**Authors:** Friederike Maechler, Frank Schwab, Sonja Hansen, Michael Behnke, Marc J. Bonten, Rafael Canton, Cristina Diaz Agero, Carolina Fankhauser, Stephan Harbarth, Benedikt D. Huttner, Axel Kola, Petra Gastmeier

**Affiliations:** 1 Institute of Hygiene and Environmental Medicine, Universitätsmedizin – Charité Berlin, Germany; 2 Department of Medical Microbiology and Infection Control, University Medical Center Utrecht, Utrecht, Netherlands; 3 Servicio de Microbiología, Hospital Universitario Ramón y Cajal and Instituto Ramón y Cajal de Investigación Sanitaria, Madrid; 4 Servicio de Medicina Preventiva y Salud Pública Hospital Universitario Ramón y Cajal and Instituto Ramón y Cajal de Investigación Sanitaria, Madrid; 5 Infection Control Program, Geneva University Hospitals and Medical School, Geneva, Switzerland

## Abstract

**Objectives:**

The aim of this study was to quantify the time delay between screening and initiation of contact isolation for carriers of extended-spectrum beta-lactamase (ESBL)–producing Enterobacterales (ESBL-E).

**Methods:**

This study was a secondary analysis of contact isolation periods in a cluster-randomized controlled trial that compared 2 strategies to control ESBL-E (trial no. ISRCTN57648070). Patients admitted to 20 non-ICU wards in Germany, the Netherlands, Spain, and Switzerland were screened for ESBL-E carriage on admission, weekly thereafter, and on discharge. Data collection included the day of sampling, the day the wards were notified of the result, and subsequent ESBL-E isolation days.

**Results:**

Between January 2014 and August 2016, 19,122 patients, with a length of stay ≥2 days were included. At least 1 culture was collected for 16,091 patients (84%), with a median duration between the admission day and the day of first sample collection of 2 days (interquartile range [IQR], 1–3). Moreover, 854 (41%) of all 2,078 ESBL-E carriers remained without isolation during their hospital stay. In total, 6,040 ESBL-E days (32% of all ESBL-E days) accrued for patients who were not isolated. Of 2,078 ESBL-E-carriers, 1,478 ESBL-E carriers (71%) had no previous history of ESBL-E carriage. Also, 697 (34%) were placed in contact isolation with a delay of 4 days (IQR, 2–5), accounting for 2,723 nonisolation days (15% of ESBL-E days).

**Conclusions:**

Even with extensive surveillance screening, almost one-third of all ESBL-E days were nonisolation days. Limitations in routine culture-based ESBL-E detection impeded timely and exhaustive implementation of targeted contact isolation.

Many guidelines^1,2^ recommend the identification of Enterobacterales that produce extended-spectrum β-lactamases (ESBL-E) among patients at risk as well as the subsequent use of contact isolation when caring for carriers (sometimes limited to specific species).

Both the effectiveness and the clinical relevance of ESBL-E screening programs have been questioned.^3,4^ Evidence regarding the likelihood of colonized ESBL-E carriers to become infected is conflicting.^5–9^ A negative screening sample does not exclude an infection caused by gram-negative bacteria that require treatment with a carbapenem,^4^ and screening with subsequent targeted contact isolation has failed to show a benefit in the endemic setting.^10–12^ Current culture-based methods are slow,^13^ and false-negative results are relatively frequent.^3^

To guide infection control interventions effectively, the turnaround time of the procedures should be as short as possible to avoid delay until the initiation of contact isolation or to avoid unnecessary isolation days in the case of pre-emptive isolation measures for patients at risk of carriage.

A large cluster-randomized trial in 20 non-ICU wards from 4 European countries reported no additional benefit of contact isolation over standard precautions to prevent new acquisition of ESBL-E.^10^ To better understand these study results, we quantified the delay between collecting the screening culture and subsequent contact isolation for carriers of ESBL-E in a post hoc analysis of the contact isolation period. More specifically, we sought to determine the proportions of ESBL-E carriers who were not in contact isolation and the ESBL-E days without contact isolation, both among patients with a previous history of ESBL-E carriage and among patients newly identified through the screening.

## Methods

### Study setting and patients

The project was a secondary analysis of the contact isolation period within a cluster-randomized crossover trial that compared contact isolation and standard precautions to control ESBL-E in 4 European countries.^10^ The patients were from 20 adult medical, surgical, or combined medical–surgical wards in Germany (8 wards), the Netherlands, Spain, and Switzerland (4 wards each).

During contact isolation periods, patients with a known history of ESBL-E were cared for under contact isolation beginning at the time of admission. For those without a history of ESBL-E, contact isolation was initiated when ESBL-E was detected in a surveillance or clinical culture. Contact isolation required accommodation of patients with ESBL-E in single-bed rooms, side rooms with spatial separation, or shared rooms with other patients with ESBL-E, as well as the use of gloves and gowns for contacts with ESBL-E–positive patients.^10^

### Microbiological cultures

Rectal swabs for ESBL-E surveillance cultures were collected on the day of admission or the following 2 days, once a week thereafter, and on the day of discharge or the prior and/or following 2 days. Swabs were processed at the local microbiological laboratories following a standardized protocol. Chromogenic agar plates were used for detection of ESBL-E (bioMérieux, Marcy l’Etoile, France). Disk diffusion, Vitek2 (bioMérieux), or MicroScan (Beckman Coulter, Brea, CA) was used to confirm ESBL-E production. Swab results were reported back to the wards as part of routine laboratory notification procedures.^10^

### Data collection and definitions

We collected data on patient admissions and discharges, surveillance samples, and ESBL-E–positive clinical cultures. Among ESBL-E–positive patients, we collected data on the history of ESBL-E carriage, the day of ESBL-E notification to the ward, and the use of barrier precautions per hospital day.

ESBL-E carriers were patients with at least 1 positive ESBL-E screening or clinical culture. We considered a patient as an ESBL-E carrier from the day the first positive culture was obtained until discharge, even if follow-up samples were negative. Acquisition of ESBL-E carriage was defined as recovery of ESBL-E isolates from clinical or surveillance cultures after hospital day 3 following an initial negative culture.

ESBL-E days were defined as the days between the collection of the first positive sample until discharge for patients both with and without history of previous ESBL-E carriage.

Days without notification were the days between collecting the first positive sample until infection control personnel informed the healthcare workers (HCWs) on the ward of the patient with the positive result. If contact isolation was initiated after the first ESBL-E positive culture was collected, but prior to the day of notification, the patient was considered as a known ESBL-E carrier on the ward from the first day of contact isolation.

Isolation days were defined as the days between the first day each ESBL-E patient was labeled under contact isolation according to routine hospital records until discharge. Patients with a known history of ESBL-E carriage were placed in pre-emptive contact isolation upon admission; pre-emptive contact isolation was discontinued when screening results were negative. We considered the days of sample collection and the first day of contact isolation as full ESBL-E and isolation days, respectively.

To describe the potential impact of time delay between collecting the samples and subsequent contact isolation before the ESBL-E carriers were discharged from the wards, we divided the ESBL-E carriers into 4 groups:

- Group 1: ESBL-E carriers known on admission were ESBL-E carriers with a history of ESBL-E carriage prior to admission. For confirmation of ESBL-E carriage, a positive admission sample was required. Contact isolation was initiated from the day of admission.
- Group 2: Same definition as for group 1, but contact isolation was not initiated during the patient’s stay on the ward.
- Group 3: ESBL-E carriers without previous carriage, who were first identified through screening or clinical samples on the wards. Contact isolation was initiated during the stay on the ward once the positive result was available.
- Group 4: Same definition as for group 3, but contact isolation was not initiated during the patient’s stay on the ward.

### Statistical analysis

The primary outcome was the proportion of nonisolated ESBL-E days among all identified ESBL-E days. Secondary outcomes were time intervals (1) between the day of admission and the day the first screening sample was collected or (2) the day the first ESBL-E–positive screening or clinical culture was obtained until the first day of contact isolation. For categorical parameters, we calculated number and percentages; for continuous parameters, we calculated median and interquartile ranges (IQRs). Wald confidence intervals were computed at the 95% level. Differences were tested by χ^2^, Kruskal-Wallis, or Wilcoxon rank-sum test. All analyses were performed with SPSS version 25, software (IBM, Chicago, IL) and SAS version 9.4 software (SAS Institute, Cary, NC).

### Ethics statement

The study protocol for the original study is available online and was approved by all local institutional review boards.^10^ The original study was registered (no. ISRCTN57648070).

## Results

Between January 2014 and August 2016, 19,122 patients were admitted to the participating wards during contact isolation periods and had a length of stay ≥2 days. At least 1 culture was collected for 16,091 patients (84.1%), yielding 32,253 samples. The median length of stay was 7 days (IQR, 4–11) among screened patients and 4 days (IQR, 3–7) among unscreened patients (*P* < .01).

Among all patients screened, 2,078 ESBL-E carriers were identified. The ESBL-E admission prevalence was 10.0% (95% confidence interval [CI], 9.6%–10.6%), with 1,572 (76%) of 2,078 ESBL-E carriers positive on admission. After day 3 and thus later than the interval predetermined for admission screening, 506 ESBL-E carriers (24%) were first detected with ESBL-E. Among the 8,613 patients screened twice, 472 (5.3%; 95% CI, 4.8%–5.8%) had new acquisition of ESBL-E; these 472 comprised 22.7% of ESBL-E cases. Data on previous admissions to the same ward were available for 2,058 ESBL-E carriers, of whom 523 ESBL-E carriers (25%) were readmitted.

**Fig. 1.**
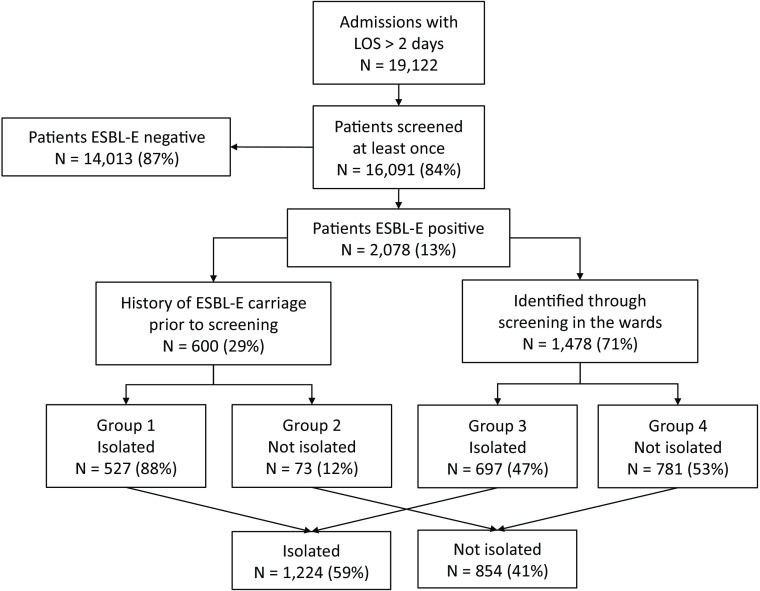
Overview of ESBL-E carriers stratified into groups of patients with history of previous ESBL-E carriage, patients newly identified through active surveillance cultures, and patients discharged before healthcare workers could be notified.

Figure 1 shows patient admissions and ESBL-E carriers stratified into groups according to HCW knowledge of the previous ESBL-E carriage. Table 1 shows the median screening delay, median delay until initiation of contact isolation, and the median number of ESBL-E and isolation days per group.

**Table 1.** Description of ESBL-E Carriers and the Isolation Compliance in Total and Stratified by Defined Groups

Variable	All ESBL-E Carriers	All ESBL-E Carriers		*P* Value	Group 1	Group2	Group 3	Group 4	*P* Value
Contact isolation status at any time during the ESBL-E carrier’s stay on the ward	…	Isolated	Nonisolated		Isolated	Nonisolated	Isolated	Nonisolated	
Identification of ESBL-E carriage	…	…	…		History of ESBL-E	History of ESBL-E	Newly identified by screening	Newly identified by screening	
Notification status of ESBL-E carriage (during stay)	…	…	…		…	…	Notification	Without notification	
Admission, no. (% of all ESBL-E carriers)	2,078 (100)	1,224 (58.9)	854 (41.1)		527 (25.4)	73 (3.5)	697 (33.5)	781 (37.6)	
Patient days, no. (% of all patient days, row %)	25,738 (100)	19,582 (76.1)	6,156 (23.9)		7,475 (29)	406 (1.6)	12,107 (47)	5,750 (22.3)	
ESBL-E days (patient days from the first positive sample), no. (% of all ESBL days, row %)	18,698 (100)	15,794 (84.5)	2,904 (15.5)		6,187 (33.1)	231 (1.2)	9,607 (51.4)	2,673 (14.9)	
Length of stay, median d (IQR)	8 (5–15)	12 (7–21)	5 (4–8)	<.001	10 (7–19)	4 (3–6)	13 (8–22)	5 (4–8)	<.001
Duration from admission to first sample, median d (IQR)	2 (1–3)	2 (1–2)	2 (1–3)	<.001	2 (1–2)	2 (1–3)	2 (1–2)	2 (1–3)	.096
Duration from admission to first positive sample, median d (IQR)	2 (1–5)	2 (1–4)	3 (2–6)	.017	2 (1–3)	2 (1–4)	2 (1–5)	3 (2–6)	<.001
Duration from first positive sample to contact isolation, median d (IQR)	2 (−1 to 5) N = 1,224	2 (−1 to 5) N = 1,224	(-) N = 0	.464	−1 (−2 to 0) N = 527	(-) N = 0	4 (2–5) N = 697	(-) N = 0	<.001
Duration from first positive sample to discharge, median d (IQR)	5 (3–11)	9 (6–17)	3 (2–4)	<.001	8 (5–16)	3 (2–4)	10 (6–17)	3 (2–4)	<.001
Patient days, no. (%)	25,738 (100)	19,582 (100)	6,156 (100)		7,475 (100)	406 (100)	12,107 (100)	5,750 (100)	
Patient days before first positive sample (ESBL-E negative days), no. (% of patient days, column %)	7,040 (27.4)	3,788 (19.3)	3,252 (52.8)		1,288 (17.2)	175 (43.1)	2,500 (20.7)	3,077 (53.5)	
ESBL-E days (patient days from the first positive sample), no. (% of patient days, column %)	18,698 (72.6)	15,794 (80.7)	2,904 (47.2)		6,187 (82.8)	231 (56.9)	9,607 (79.4)	2,673 (46.5)	
ESBL-E days (patient days from the first positive sample), no. (% of ESBL days)	18,698 (100)	15,794 (100)	2,904 (100)		6,187 (100)	231 (100)	9,607 (100)	2,673 (100)	
ESBL-E days in contact isolation, no. (% of ESBL-E days, column %)	12,658 (67.7)	12,658 (80.1)	0 (0)		5,774 (93.3)	0 (0)	6,884 (71.7)	0 (0)	
ESBL-E days without contact isolation, no. (% of ESBL-E days, column %)	6,040 (32.3)	3,136 (19.9)	2,904 (100)		413 (6.7)	231 (100)	2,723 (28.3)	2,673 (100)	

Among the 2,078 ESBL-E carriers, 1,224 (59%) were placed in contact isolation at some time during their stay on the ward. The median lengths of stay were 12 days (IQR, 7–21) among isolated ESBL-E carriers and 5 days (IQR, 4–8) among nonisolated ESBL-E carriers.

**Fig. 2.**
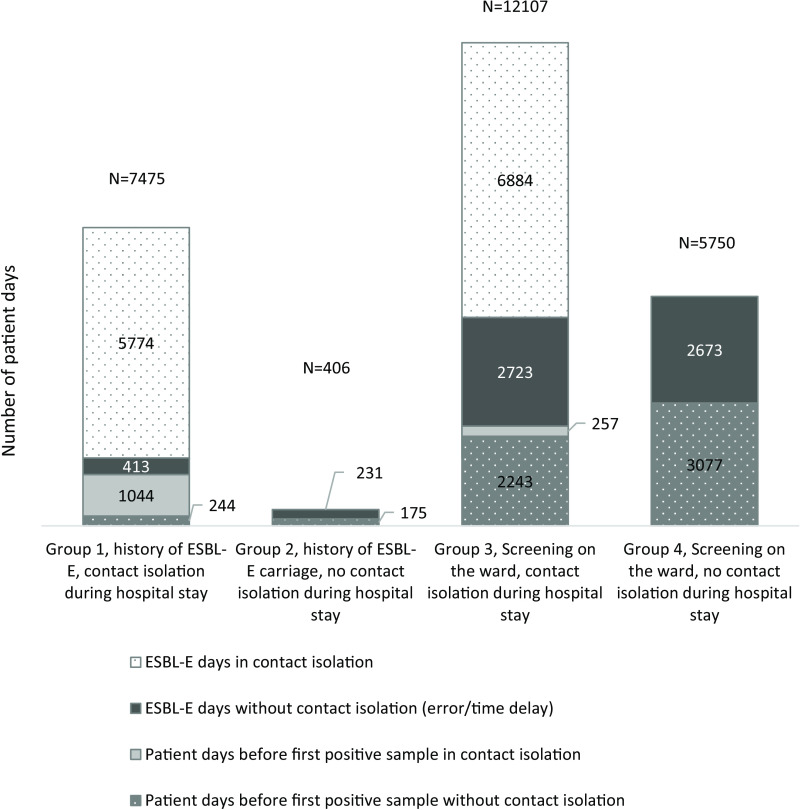
Number of patient days before the first positive sample and identified ESBL-E days with and without contact isolation, stratified by ESBL-E carrier group.

ESBL-E carriers identified through screening cultures in time for subsequent contact isolation [group 3, comprising 697 (34%) of all ESBL-E carriers] accounted for 9,607 (51%) of 18,698 ESBL-E days. Because of the delay between collecting the sample and initiation of contact isolation, 28% of those were nonisolated ESBL-E days [2,723 (15%) of 9,607 ESBL-E days] (Table 1 and Fig. 2). Among these newly identified ESBL-E patients (ie, group 3), contact isolation was initiated on a day later than the day the patient was first reported positive to the wards by the infection control staff for 63 (9.6%) of 650 ESBL-E patients (with complete data), accounting for 127 ESBL-E days with information on the patient’s ESBL-E carriage but without contact isolation.

The median durations between collecting the positive sample and initiation of contact isolation were 4 days IQR, 2–5 days) among patients without a prior history of ESBL-E carriage, both among the 1,006 previously unknown ESBL-E carriers positive on admission and 4 days IQR, 3–6 days) among the 472 ESBL-E carriers who were first identified during their stay on the ward. Supplementary Table S1 in the Supplementary Appendix (online) shows the screening compliance, length of stay, ESBL-E days, and ESBL-E days in contact isolation per institution and site.

**Fig. 3.**
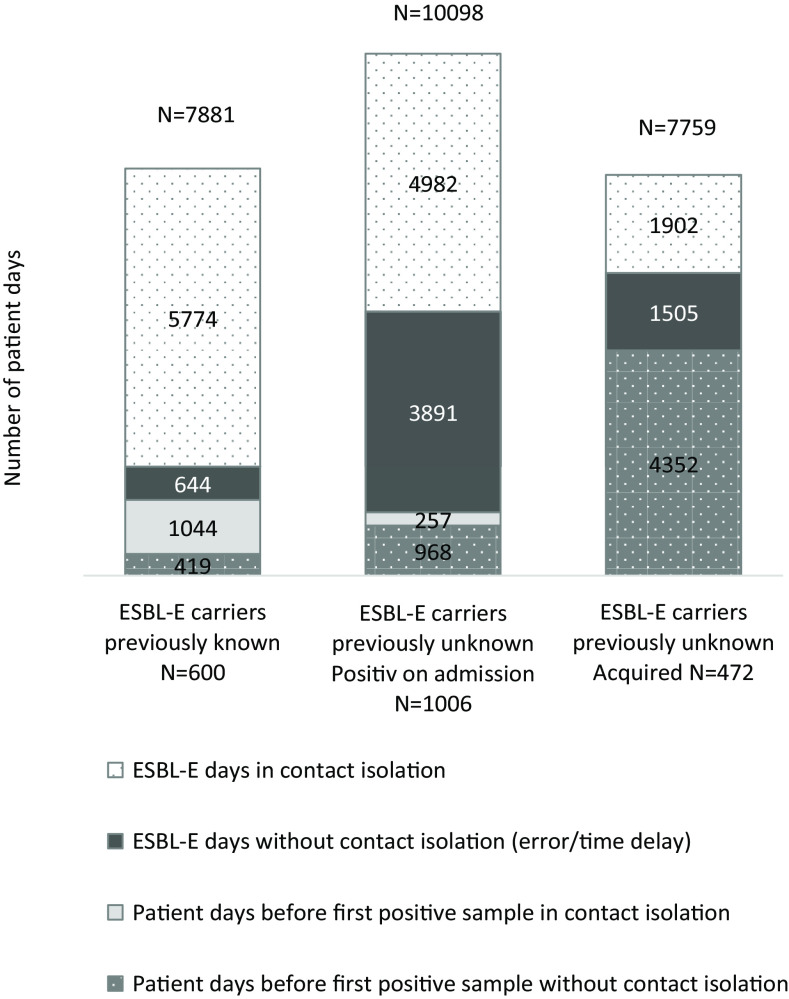
Percentage and number of patient days identified ESBL-E days with and without contact isolation by ESBL-E carrier status.

Figure 2 shows the percentages and numbers of patient days with and without ESBL-E carriage and with and without contact isolation for groups 1, 3, and 4. Figure 3 shows the ESBL-E days and isolation days used for ESBL-E carriers who were positive on admission or who acquired ESBL-E on the wards.

## Discussion

In this secondary analysis of a large clinical trial in four European healthcare institutions with endemic ESBL-E, despite using an extensive screening program exceeding routine hospital practice, surveillance cultures obtained on the study wards identified only little more than one-third of ESBL-E carriers in time for subsequent contact isolation. Moreover, 41% of ESBL-E carriers were discharged before any pathogen-directed preventive measures could be started.

Debate continues regarding the value of surveillance screening for ESBL-E.^14^ Infection prevention measures are often categorized into vertical and horizontal strategies. Vertical strategies target a specific organism and often include active surveillance cultures followed by isolation practices, whereas horizontal strategies aim to control the spread of multiple organisms simultaneously.

Screening as part of a vertical strategy to prevent the spread of ESBL-E should only be undertaken if identified ESBL-E carriers are treated differently from non–ESBL-E carriers. Otherwise, there would be no added value over horizontal strategies.

Several factors may explain the limitations of approaches involving screening for ESBL-E carriage. First, in our cohort, the median duration between collecting the positive sample and initiation of contact isolation was 4 days among patients without previous history of ESBL-E carriage. Laboratory procedures may be faster in other settings, or may have accelerated since,^15^ but we used routine chromogenic culture-based procedures and laboratories in 4 European university hospitals at the time the study was conducted. Day-wise instead of hourly data collection may have overestimated turnaround time. Other groups have reported the turnaround time to identify positive ESBL-E to be 4 days for positive ESBL-E cultures on selective screening media followed by antimicrobial susceptibility testing (as opposed to 1 day for negative cultures).^13^ During that time, contact isolation will either have to be delayed or must be applied to all patients instead.^16^ Notably, even total laboratory automation could not significantly reduce the median turnaround time for positive ESBL-E samples.^16^ Time delay also encompasses the time between collecting the sample, transporting it to the laboratory, reporting the results back to the infection control team and/or directly to the HCWs, until the initiation of contact isolation. Improving the turnaround time from collecting the specimen until initiating any intervention based on the screening result will thus require rapid and efficient preanalytic, analytic, and postanalytic processes both within and outside the laboratory.

The analytical laboratory processes could be considerably shortened with the use of rapid molecular techniques because total hands-on time in the laboratory from the arrival of the sample to the test result rarely exceeds 1 hour.^17^ Molecular methods may even provide an advantage in terms of screening sensitivity because the low level of resistance conferred by some genes sometimes leads to a lack of phenotypic detection, which only appears at a clinically significant level under antibiotic selective pressure.^17^ On the other hand, molecular methods may lack clinical relevance because they detect both viable or nonviable microorganisms.^17^ Currently, there are no biochemical or PCR screening methods for general routine testing practice that are comprehensive, reliable, and rapid.^17^ Many molecular assays rely on the detection of only 1 or 2 genes, making their use limited in settings where multiple antimicrobial resistance genes are prevalent. A good knowledge of the local gene pool is therefore important before implementing a molecular method.^18^

Second, screening approaches on admission in real-life scenarios are often hindered by time delays between admission and collecting the sample, particularly in non-ICU wards, because patients are often mobile and may thus not be present during screening rounds. Moreover, discharge management and completion of nonessential examinations may not always align perfectly. Our results show that patients without screening had a shorter LOS compared with screened patients. This difference in length of stay was even more striking in patients with and without contact isolation, reflecting the combined effect of incomplete adherence to screening requirements, a relatively long turnaround time and subsequent contact isolation in patients with a short LOS.

Third, routine hospital-based MDRO surveillance strategies only rarely comprises screening cultures obtained after admission to the wards. ESBL-E acquisition within the hospital, for example, through patient-to-patient transmission or through selection processes by antimicrobial chemotherapy^19^ or therapeutic drugs other than antimicrobials,^20^ cannot be detected by admission screening programs. In our study, >20% of all ESBL-E carriers and almost 20% of ESBL-E days would have been missed by a screening protocol based solely on admission cultures, and other projects have reported similar findings.^19^

Almost 30% of ESBL-E carriers had a history of ESBL-E carriage; among them, almost 90% were placed in contact isolation from their first day of admission. Therefore, the benefit of screening programs should be considered not only for the patient’s current hospital stay during which the culture was obtained but also for potential future readmissions. Following this line of argument, in terms of subsequent hospital admissions, systematic discharge screening would be even more relevant.

Fourth, even a universal screening protocol focusing only on admissions generates massive workload for healthcare and laboratory staff as well as substantial expenditures for the hospital system, whereas its health–economic benefit has not been evaluated in the contemporary epidemiological context.^14^ On the other hand, although targeted screening of patients with prespecified risk factors for ESBL-E carriage could offer a cost-saving alternative,^21^ a reliable risk assessment for ESBL-E carriage upon hospital admission appears still not possible.^22,23^ Thus, surveillance screening could miss some ESBL-E carriers who could then serve as unknown reservoirs of ESBL-E and facilitate spread within the healthcare facility.

The dilemma between increasing burdens on patients and institutions in a rather imperfect attempt to prevent further spread or accepting the ineffectiveness of the screening program is yet to be solved.^24^ A time delay of 4 days between collecting the sample and initiation of contact precautions for ESBL-E patients identified through culture-based screening on the ward certainly affects the effectivity of contact isolation. However, universal screening strategies with neither conventional^10^ nor rapid molecular methods^12^ followed by subsequent contact isolation precautions for identified carriers were successful in reducing ESBL-E incidence, both in the ICU and the non-ICU endemic setting. Not even pre-emptive isolation precautions for all patients admitted to the wards until the patients were proven negative showed a beneficial effect for reducing the spread of intestinal multidrug-resistant bacteria,^25^ although this has not yet been shown for ESBL-E. Considering these limitations of screening strategies, and that an increase of patients in contact isolation may actually lead to a decrease of adherence to the contact precautions,^26^ the debate regarding whether or not contact isolation precautions would be more effective if only all ESBL-E carriers were identified in time may be an academic one.

This study had several limitations. Adherence to the screening protocol was not 100%, but the screening extended well beyond what could be expected from real-life scenarios. We may have underestimated the number of ESBL-E days because the screening protocol allowed for admission screening within the first 3 days of admission (day of admission plus the following 2 days). We were unable to provide more granularity on the specific cause of the time delay because we did not collect data on the arrival time of the specimen in the laboratory or the timestamp of the laboratory result. In addition, the aforementioned day-wise instead of hourly data collection may have overestimated turnaround time. We screened surveillance cultures from rectal swabs without broth enrichment and extended incubation to enhance test sensitivity because turnaround time for reporting positive results would have increased.^27^

Despite maximum efforts in terms of surveillance screening, initiation of contact isolation was not possible for a large proportion of ESBL-E carriers before discharge. Although the screening protocol far exceeded routine practice, and although the samples were processed by local laboratories from 4 European university hospitals, almost one-third of all ESBL-E days were nonisolated, and >40% of ESBL carriers were not placed in contact isolation precautions while on the ward. As long as limitations in routine ESBL-E detection as well as high numbers of ESBL-E carriers impede timely, exhaustive, and correct implementation of targeted infection control approaches, efforts to contain the spread of ESBL-E within the hospital should focus on horizontal approaches such as standard precautions and antibiotic stewardship.

## Supplementary material

For supplementary material accompanying this paper visit https://doi.org/10.1017/ice.2022.285. click here to view supplementary material
