# Supplementary material for: Quantification of time delay between screening and subsequent initiation of contact isolation for carriers of extended-spectrum beta-lactamase (ESBL)–producing Enterobacterales: A post hoc subgroup analysis of the R-GNOSIS WP5 Trial
Source: Infect Control Hosp Epidemiol. 2023 Mar 13;44(9):1410–6. doi: 10.1017/ice.2022.285 (PMC10507499; doi:10.1017/ice.2022.285)
Supplement: Supplementary file 1 [file S0899823X22002859sup001.docx]

# Supplementary Material

Table S1: Overview of adherence to screening, length of stay (LOS) and days with carriage ESBL-producing Enterobacterales per institution and per ward

|  | **Site** | **Screening, %** | **LOS**  **(days), N** | **ESBL-E**  **Days, N** | **ESBL-E days**  **in CI, N** | **ESBL-E days**  **in CI, %** | **ESBL-E days**  **of PD, %** |
| --- | --- | --- | --- | --- | --- | --- | --- |
| **Total** | total | 84 | 9.3 | 18698 | 12658 | 68% | 13% |
| **Institution** | A | 80 | 8.4 | 8890 | 5997 | 67% | 14% |
|  | B | 82 | 15.7 | 3599 | 2793 | 78% | 17% |
|  | C | 93 | 8.6 | 3666 | 2229 | 61% | 9% |
|  | D | 84 | 9.5 | 2543 | 1639 | 64% | 11% |
| **Wards** | 1 | 90 | 7.9 | 1175 | 698 | 59% | 12% |
|  | 2 | 72 | 19.9 | 503 | 330 | 66% | 11% |
|  | 3 | 62 | 5.2 | 377 | 116 | 31% | 9% |
|  | 4 | 81 | 6.7 | 888 | 375 | 42% | 8% |
|  | 5 | 70 | 12.8 | 1790 | 1431 | 80% | 27% |
|  | 6 | 83 | 10.0 | 1266 | 1011 | 80% | 15% |
|  | 7 | 85 | 8.4 | 1639 | 1145 | 70% | 17% |
|  | 8 | 87 | 8.5 | 1252 | 891 | 71% | 12% |
|  | 9 | 68 | 8.6 | 617 | 336 | 54% | 13% |
|  | 10 | 95 | 15.7 | 1567 | 1329 | 85% | 24% |
|  | 11 | 99 | 26.3 | 693 | 578 | 83% | 15% |
|  | 12 | 100 | 25.6 | 722 | 550 | 76% | 13% |
|  | 13 | 95 | 9.5 | 1214 | 735 | 61% | 10% |
|  | 14 | 96 | 9.0 | 909 | 494 | 54% | 8% |
|  | 15 | 91 | 6.6 | 890 | 475 | 53% | 9% |
|  | 16 | 87 | 12.0 | 653 | 525 | 80% | 11% |
|  | 17 | 58 | 8.4 | 328 | 192 | 59% | 14% |
|  | 18 | 89 | 9.6 | 906 | 592 | 65% | 12% |
|  | 19 | 90 | 10.3 | 439 | 269 | 61% | 6% |
|  | 20 | 89 | 9.0 | 870 | 586 | 67% | 13% |

LOS is length of stay, ESBL-E are Enterobacterales with production of extended spectrum betalactamase, CI is contact isolation, PD are patient days
